# Corneal endothelial ring following the implantation of toric implantable collamer lenses with a central hole: a case report

**DOI:** 10.1186/s12886-022-02712-4

**Published:** 2022-12-16

**Authors:** Jun Zhu, Qi Dai, Yu-feng Ye

**Affiliations:** Eye Hospital of Wenzhou Medical University, 618 East Fengqi Road, 310000 Hangzhou, Zhejiang China

**Keywords:** Corneal endothelial ring, Air-puff tonometry, TICL V4C, Case report

## Abstract

**Background:**

To report a case of a corneal endothelial ring after toric implantable collamer lens (TICL, V4C) implantation in the right eye of a patient.

**Case presentation:**

A 36-year-old woman with refractive errors of -8.00 DS/-2.00 DC * 8° in the right eye and − 6.50 DS/-1.75 DC * 177° in the left eye developed a corneal endothelial ring in the right eye on the first day after receiving TICLs implantation for treatment of high myopic astigmatism, which has not been previously reported as a complication of ICLs implantation. At 1 day postoperatively, the uncorrected distance visual acuity (UDVA) was 20/16, the intraocular pressure as measured by non-contact tonometry was 16.9 mmHg, and the vault as measured by anterior segment optical coherence tomography was 1238 μm. The eye was quiet and there was no unusual anterior chamber reaction. However, slit-lamp examination revealed an endothelial annular lesion of approximately 0.4 mm in diameter in the central part of the cornea, which was gray-white in color. The shape of the ring was the same as that of the central hole of the TICL. Specular microscopy showed that the mean endothelial cell density (ECD) of the ring significantly decreased to 1442 ± 263 cells/mm^2^, while the other part was still normal (2852 ± 103 cells/mm^2^). After 9 days of corticosteroid treatment and intense lubrication, the patient had a clear cornea, increased ECD (1532 ± 653 cells/mm^2^), and a good UDVA (20/16).

**Conclusion:**

This case suggests that a few hours after ICL V4C implantation, with a large vault, corneal displacement caused by an air puff would make the endothelium close to or even contact the ICL, producing a corneal endothelial ring. After ruling out various possible factors, we speculated that the endothelial ring was developed due to the non-contact tonometer air puff before slit-lamp evaluation, and this phenomenon was recorded by Corvis, which confirmed that the cornea could come in contact with the ICL due to gas shock. This “contact” may cause transient corneal endothelial damage.

**Supplementary Information:**

The online version contains supplementary material available at 10.1186/s12886-022-02712-4.

## Background

Implantable collamer lens (ICL) implantation is a widely accepted refractive surgery for correction of high myopia and myopic astigmatism when the corneal laser ablation procedure is not suitable for patients [1, 2]. ICL implantation has shown promising long-term results in terms of fast visual recovery, stable refraction, and reversibility [3]. Although considered safe, ICL implantation may be associated with complications such as early or late cataractogenesis, postoperative intraocular pressure (IOP) elevation, retinal detachment, and corneal endothelial cell failure [4].

To our knowledge, most reported cases of corneal endothelial rings, known as traumatic corneal endothelial rings (TCERs), are caused by blunt ocular trauma. There have been no reports of corneal endothelial rings as a complication of ICL implantation. Herein, we discuss a patient who developed a gray-white corneal ring in the right eye after ICL implantation.

### Case presentation

A 36-year-old woman diagnosed with myopia presented for the evaluation of refractive surgery. She was a teacher with no history of systemic or ocular disease or surgery.

The anterior segment and fundus evaluations, including slit-lamp bio-microscopy and indirect ophthalmoscopy (+ 90 D lens), were normal in both eyes. The patient’s uncorrected distance visual acuity (UDVA) was 1/20 in both eyes, with refractive error of -8.00 DS/-2.00 DC * 8° in the right eye and − 6.50 DS/-1.75 DC * 177° in the left eye. IOP was 15.9 and 15.2 mmHg in the right and left eye, respectively, measured using applanation tonometry (TX-F; Canon, Tokyo, Japan). The corneal topography was measured using a rotating Scheimpflug camera (Pentacam HR, Oculus, Germany). Keratometric values were 43.5@100/41.5@10 and 43.2@92/41.3@2, the anterior chamber depth (ACD) was 3.35 and 3.37 mm, central corneal thickness (CCT) was 505 and 501 μm for the right and left eye, respectively. White-to-white (WTW) distance was 11.9 mm for both eyes, while WTW measured using Zeiss IOL-Master 700 was 12.2 mm for both eyes. Horizontal sulcus-to-sulcus (STS) distance assessed by the UBM (Ultrasound Biomicroscope, Model SW-3200 L; Tianjin Suowei Electonic Technology Co, Ltd., Tianjin, China) were 12.28 mm and 12.13 mm for the right and left eye, respectively. The endothelial cell density (ECD), calculated by the non-contact autofocus specular microscope (EM-3000, Tomey Corp., Nagoya, Japan), was 2664 cells/mm^2^ in the right eye and 2557 cells/mm^2^ in the left eye (automatic method was used for endothelial count analysis).

After a thorough discussion with the patient regarding the risks and benefits of surgery, we obtained informed consent for surgery.

Toric ICL-V4Cs (Visian ICL with Centraflow; STAAR Surgical) were chosen for horizontal implantation, with a power of -10.50 DS/+ 2.00 DC * 99° and 1° clockwise rotation in the right eye and − 9.00 DS/+ 1.50 DC * 88° and 1° clockwise rotation in the left eye (VTICMO13.2 SN: T564197 for the right eye, VTICMO13.2 SN: T557998 for the left eye).

Preoperatively, 0° and 180° limbus reference markings were made under a slit lamp, and the surgery was performed under pupil dilation. After administration of topical anesthesia (0.5% proparacaine hydrochloride eye drops; Ruinian Best, Nanjing, China, with no preservatives), two side-ports (1.0 mm in size) and a 3.0 mm vertical clear corneal main incision were made. One side-port incision was used for continuous infusion of balanced salt solution (BSS) [5] by the patent irrigator to maintain the anterior chamber, and the other was used for tucking the footplates by the patent manipulator. The TICL was inserted through the main incision with four haptics tucked beneath the iris, and subsequently adjusted to the desired alignment axis. No ophthalmic viscosurgical devices (OVDs) were used during surgery.

The patient was discharged two hours after bilateral TICLs implantation, with a clear cornea, mid-dilated pupil, and UDVA of 6/20 and 10/20. The IOP was 28 and 25 mmHg in the right and left eye, respectively; therefore, the patient was called for follow-up the next day.

**Fig. 1:**
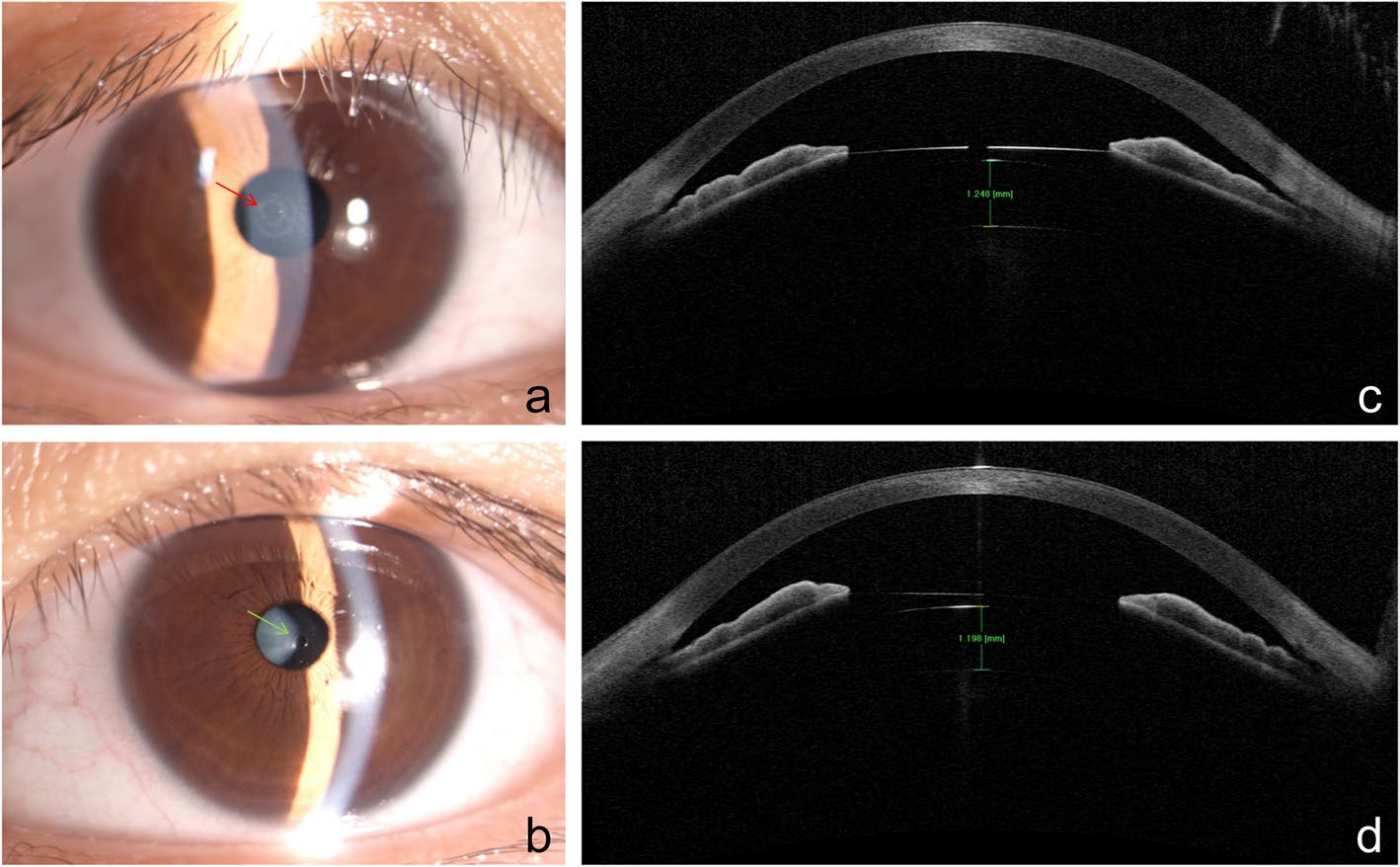
Slit-lamp photographs and AS-OCT images of both eyes (**a** to **d**). **a**,** b** The slit-lamp photographs, the red arrow indicates an endothelial annular lesion of approximately 4 mm in diameter, gray-white in right eye, while the green arrow is showing the central hole of the TICL V4C. The AS-OCT images displayed the vault of both eyes (**c** to **d**). AS-OCT, anterior segment optical coherence tomography; TICL, toric implantable collamer lens

**Fig. 2:**
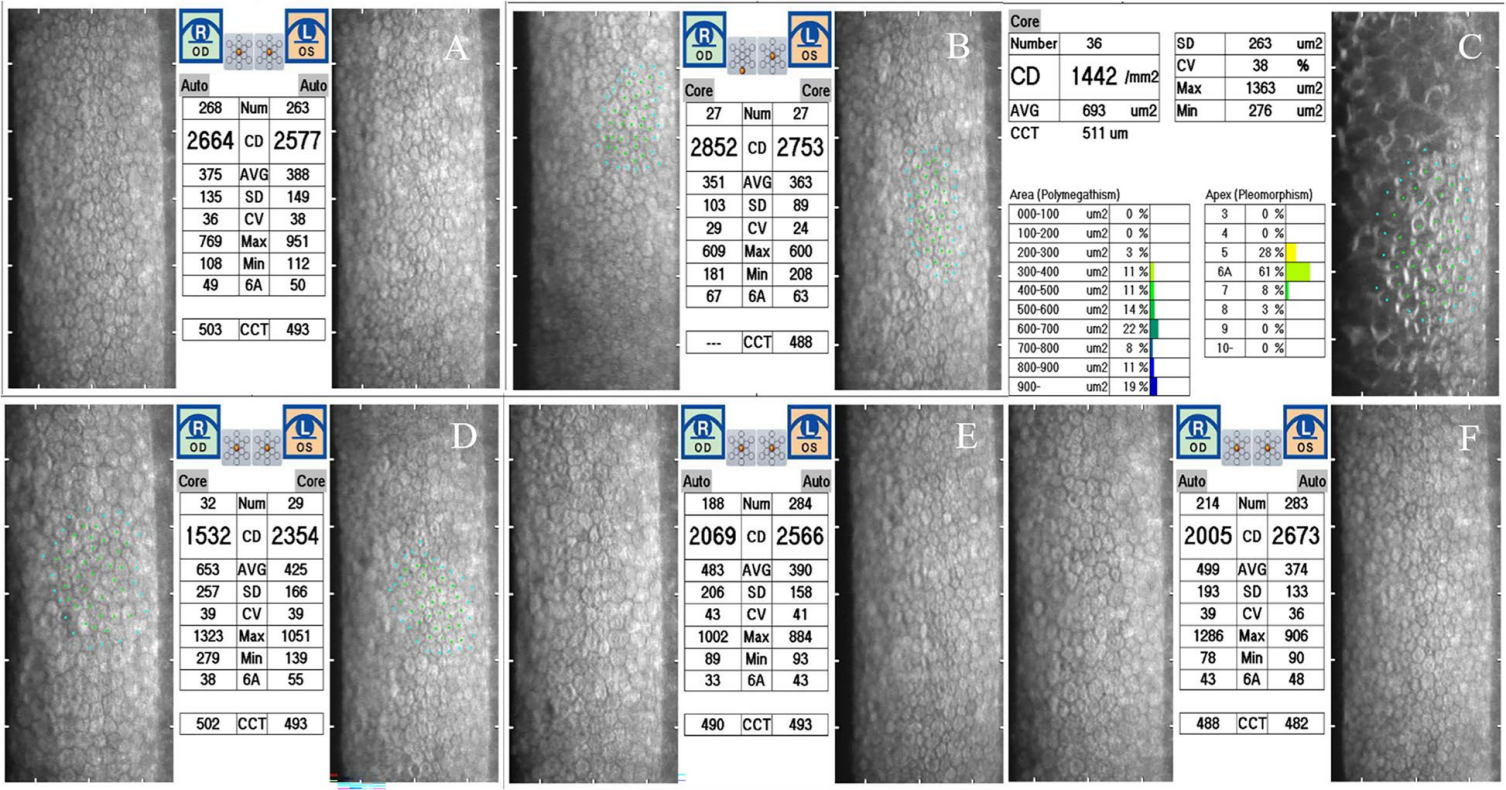
Specular microscopy image of mean ECD and the cornea endothelial cells with normal morphology at different stages. **A** Before the operation, the mean ECD of the central part of the right and left eye was 2664 ± 375 cells/mm2 and 2577 ± 388 cells/mm2, respectively. **B** On the 1st postoperative day, the mean ECD in the peripheral part of the right cornea was 2852 ± 103 cells/mm2, while in the central part of the left cornea was 2753 ± 363 cells/mm2. **C** Image showing the mean ECD of the ring (at the central part of the right cornea) was significantly decreased to 1442 ± 263 cells/mm2 with abnormal endothelial cell morphology. **D** On the 9th postoperative day, the mean ECD of the central part of the right cornea was increased to 1532 ± 257 cells/mm2. However, partial endothelial cell loss was still observed, but with better endothelial cell morphology. **E**-**F** At 4-month postoperatively, the mean ECD of the central part of the right cornea was increased to 2069 ± 483 cells/mm2 and 2005 ± 499 cells/mm2, respectively. ECD, endothelial cell density

On the first postoperative day, both eyes achieved a UDVA of 20/16 with IOP of 16 and 19 mmHg, while the vault was 1238 and 1198 μm for the right and left eye, respectively. Anterior segment examination revealed an endothelial annular lesion of approximately 0.4 mm in diameter in the central part of the cornea, gray-white in color (Fig. 1), in the right eye. The epithelium was intact without any damage. The anterior chamber was quiet without signs of anterior ocular chamber inflammation. Automatic specular microscopy showed that the mean ECD of the ring was significantly decreased to 1442 ± 263 cells/mm^2^ with abnormal endothelial cell morphology in the lesion part, whereas in the peripheral part of the cornea, it was 2852 ± 103 cells/mm^2^ (Fig. 2).

**Fig. 3:**
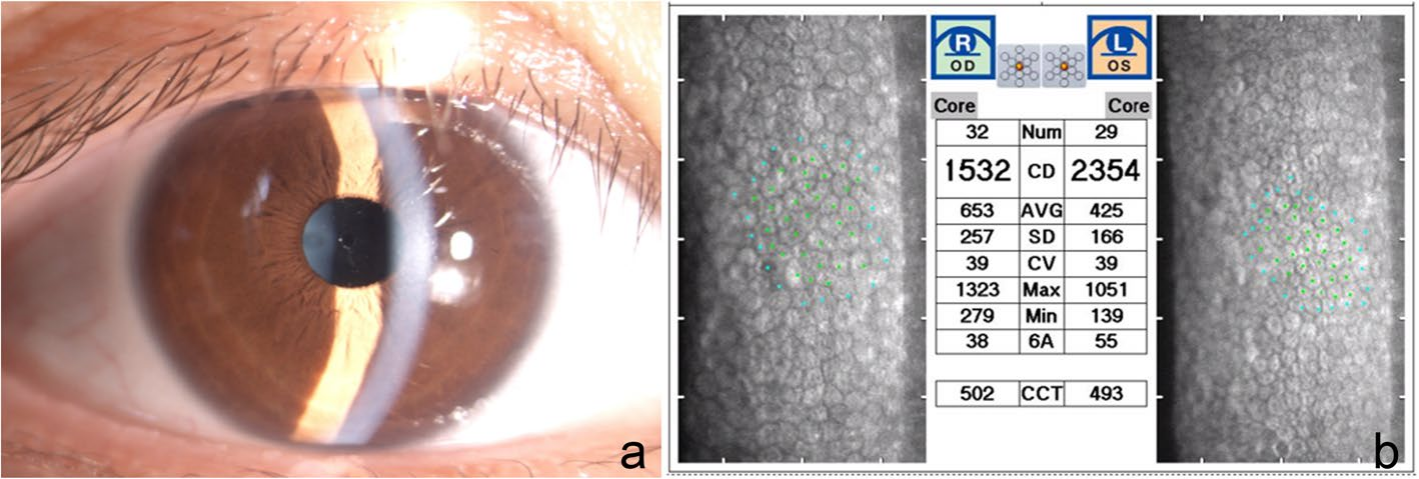
Slit-lamp photographs and specular microscopy images of the right eye, 9 days after surgery. **a** The slit-lamp photograph showed clear cornea, **b** indicating the mean ECD at central part was increased to 1532 ± 257 cells/mm2 ECD, endothelial cell density

The patient was instructed to apply tobramycin and dexamethasone eye drops (ALCON, Novartis Pharma NV, Belgium), 6 times a day, and pranoprofen (Senju, Pharmaceutical Co. Ltd., Japan), 4 times a day, postoperatively. She was also administered vitamin A palmitate eye gel (Novartis Ophthalmics AG, Switzerland), 4 times a day, and 0.3% sodium hyaluronate eye drops (Santen Pharmaceutical Co. Ltd., Japan), 6 times a day, to maintain the tear film and a regular ocular surface. At the 9th day of follow-up for the right eye (in both eyes), the UDVA was 20/16, and the cornea was clear; the mean ECD of the central part increased to 1532 ± 257 cells/mm^2^ (Fig. 3). In addition, a partial endothelial cell loss was still observed, but with a better endothelial cell morphology. IOP and vault for the right and left eyes were 16 and 13 mmHg and 1162 and 1083 μm, respectively.

Topical dexamethasone tobramycin was replaced with 0.1% fluorometholone 10 days postoperatively and then tapered slowly over the next 3 weeks (1-day, 9-day, 40-day, 4-month, and 9-month postoperative data of the right eye are presented in Table 1, CCT changes measured by two different instruments are shown in Table 2).

**Table 1:** Postoperative data

	**Manifest refraction**	**UDVA**	**IOP(mmHg)**	**Vault(um)**	**CCT(um)**	**ECD(cells/mm**^**2**^**)**
1 day	pl	1.2	16.9	1238	519	1442
9 day	+0.25DS	1.2	16.6	1162	505	1532
40 day	+0.25DS	1.2	12.5	1212	504	2069
4 month	+0.25/-0.25*5	1.2	13.1	1178	508	2005
9 month	0/-0.50*5	1.2	15.9	1019	501	2317

*UDVA* Uncorrected distance visual acuity, *IOP* Intraocular pressure, *CCT* Central corneal thickness, *ECD* Endothelial cell density

**Table 2:** Postoperative CCT data for two eyes

	**OD-CCT(um)**		**OS-CCT(um)**	
	**Pentacam**	**EM-3000**	**Pentacam**	**EM-3000**
1 day	519	511	512	488
9 day	505	502	499	493
40 day	504	490	504	493
4 month	508	488	503	482
9 month	501	502	505	494

*CCT* Central corneal thickness

## Discussion and conclusions

To the best of our knowledge, this is the first published case of corneal endothelial ring phenomenon after TICL implantation surgery without OVDs. Corneal edema and endothelial cell loss occur during intraocular surgery as a result of the mechanical damage to the endothelium at the time of surgery [6]. We carefully checked the surgical video to ensure that the instruments used during surgery did not touch the central part of the corneal epithelium. The only thing identified was that the annulus of endothelial cells was dysfunctional and presented a gray-white ring, which was same as the central hole of the implanted TICL-V4C.

Considering the location and shape of the damage, we speculated that there must have been some kind of “contact”. There was nothing except for the aqueous humor and the implanted TICL lens in the anterior chamber. We used conventional BSS as intraocular irrigation solution, without the addition of antimicrobials, dilatants, or hemostatic agents. The trajectory of the aqueous humor movement is known to be “a gush and a partial reflux” [7]; however, this “tide phenomenon” was not strong enough to leave a narrow ring in the cornea. Hence, the “contact” might have happened between the anterior surface of TICL lens and the corneal endothelium.

Interestingly, Huang et al. found an exact contact between the corneal endothelium, iris, and lens in a 61-year-old patient using air-puff tonometry [8]. Using an applanation-type tonometer, IOP was measured by deforming the eye by applying force to the outside of the globe after surgery [9, 10]. We suspect this “contact” could happen between the corneal endothelium, iris, and ICL during air-puff tonometry, and leave a dysfunctional corneal endothelial cell ring of approximately 0.4 mm in diameter, which was nearly the same size as the central hole of ICLs (0.36 mm in diameter) [4]. We also observed the approach of the corneal endothelium and the ICLs in postoperative patients, and sometimes it looked as if there was little space between the two (Additional file 1).

Fortunately, by using intensive steroid therapy, corneal edema was reduced, and healthy endothelial cells migrated to replace cells that were dysfunctional; the cornea endothelial lesion was self-limiting and disappeared within 9 days.

In endothelial corneal rings cases, injury to endothelial cells result from damage caused by mechanical stretching of the cornea. Corneal displacement produces radial tension that is transmitted to damage delicate endothelial cells [11]. This “shock wave” is very common in published cases of TCERs [11]. We suspect that the air puff would also produce a bell-shaped propagation concussive force, which is transmitted through all layers of the cornea and subsequently produces transient endothelial cell dysfunction. Maloney et al. reported endothelial cell loss with traumatic endothelial rings, which is similar to our findings. Gerard et al. showed a TCER case with no epithelial defect [12], which was followed up for 9 days; the ring had completely resolved clinically, and the VA was 6/9 in the affected eye. These two processes were perhaps similar since we proposed that endothelial touch likely amounted to trauma. Low-energy, non-penetrating ocular blast injuries have been characterized as causing transient, gray-white endothelial ring-shaped opacities [13]. Mahiul et al. reported a traumatic corneal endothelial ring due to accidental occupational air blast injury at high velocity [13], which is similar to the “air-puff” method.

In our case, the exact reason behind this unusual sign was not clear; however, the impact of the air puff by the tonometer on the corneal epithelium could be a possibility. This suggests that a few hours after ICL-V4C implantation, with the large vault, corneal displacement caused by an air-puff would make the endothelium close to or even contact the ICL. This concussive force or “contact” may cause transient corneal endothelial damage. IOP elevation and its effects on corneal endothelial cells are important after almost all intraocular surgeries, especially ICLs implantation. This unique case illustrates that more attention must be paid to injury to the corneal endothelium when using non-contact tonometry. Further work, including a larger sample size, is necessary to elucidate the actual mechanism.

## Supplementary Information

**Additional file 1.**

## Data Availability

All data generated and analyzed during this study are included in this published article. Data and material are available from the corresponding author Yu-Feng Ye (E-mail address: 15906653199@eye.ac.cn).

## Abbreviations

TICL: Toric implantable collamer lens
ICL: Implantable collamer lens
UDVA: Uncorrected distance visual acuity
ECD: Endothelial cell density
IOP: Intraocular pressure
TCERs: Traumatic corneal endothelial rings
ACD: Anterior chamber depth
CCT: Central corneal thickness
WTW: White-to-white
STS: Sulcus-to-sulcus
BSS: Balanced salt solution
OVDs: Ophthalmic viscosurgical devices
AS-OCT: Anterior segment optical coherence tomography
UBM: Ultrasound biomicroscope
